# Measuring access to culturally appropriate food and associations with diabetes among Asian Americans and Native Hawaiians or Pacific Islanders

**DOI:** 10.1017/S1368980025101444

**Published:** 2025-11-12

**Authors:** Rujia Xie, Yanjia Cao, Jiue-An Yang, Calvin P. Tribby, Clara Voong, Marta M. Jankowska

**Affiliations:** 1 Herbert Wertheim School of Public Health and Human Longevity Science, University of California, San Diego, La Jolla, CA, USA; 2 Division of Nutritional Sciences, Cornell University, Ithaca, NY, USA; 3 Department of Geography, The University of Hong Kong, Pok Fu Lam, Hong Kong SAR; 4 Population Sciences, Beckman Research Institute, City of Hope, Duarte, CA, USA

**Keywords:** Access to healthy foods, Metabolic diseases, Health status disparities, Social determinants of health, Built environment, Crowdsourcing, Minority health

## Abstract

**Objective:**

The Food Access Research Atlas (FARA) is a common measure of food access developed by the US Department of Agriculture. However, its sole focus on supermarkets lacks specificity for culturally appropriate food. This ecological study assesses the relationship of FARA and our novel Yelp-based Ethnic Store Measure with diabetes rates among Asian Americans and Native Hawaiians or Pacific Islanders (AAPI).

**Design:**

This study leveraged crowd-sourced Yelp data to develop six culturally appropriate food access variables and compare them with FARA at the census tract (CT) level. Estimates of CT AAPI diabetes rates were calculated from hospital and emergency department discharge data. Spatial trends were examined visually and with Moran’s *I*. Ordinary least squares (OLS) and spatial lag regression (SLR) models assessed covariate-adjusted z-score standardised associations between food access measures and AAPI diabetes rates.

**Setting:**

San Diego, California, USA

**Subjects:**

All 626 CT in San Diego, CA

**Results:**

Most food access variables showed strong spatial autocorrelation. The FARA measure – percent of AAPI population beyond 0·5 miles of a supermarket – was positively associated with AAPI diabetes in OLS (1·88; 95 % CI 0·76, 3·0; *P* = 0·001) but not SLR. Our Yelp-based variable – number of AAPI restaurants per AAPI population – was negatively associated with AAPI diabetes in both OLS (–1·15; 95 % CI –2·17, –0·13; *P* = 0·03) and SLR (–1·32; *P* = 0·004). No other variables were significantly associated with diabetes.

**Conclusion:**

Area-level, culturally sensitive measures of food access offer valuable insights into the role of culturally appropriate food access on cardiometabolic health among racial and ethnic minorities.

More than one in ten (11·3 %) Americans have diabetes, of which 90–95 % are type 2 diabetes, and 38 % of US adults have prediabetes^(1)^. Diabetes is the eighth leading cause of death in the USA, with complications such as CVD, kidney disease, neuropathy, blindness and lower-extremity amputation^(1,2)^. In the USA, there are racial disparities in diabetes prevalence. Asian Americans (as defined by the US Office of Management and Budget as ‘individuals with origins in any of the original peoples of Central or East Asia, Southeast Asia, or South Asia, including, for example, Chinese, Asian Indian, Filipino, Vietnamese, Korean, and Japanese’) have a diabetes prevalence of 14·9 % and an undiagnosed diabetes rate of 5·8 %, which are 1·25 and 3 times higher than among non-Hispanic White (NHW) Americans, respectively^(3,4)^. Native Hawaiians or Pacific Islanders (defined as ‘individuals with origins in any of the original peoples of Hawaii, Guam, Samoa, or other Pacific Islands, including, for example, Native Hawaiian, Samoan, Chamorro, Tongan, Fijian, and Marshallese’) have a 2·5-fold higher likelihood of diabetes diagnosis compared to NHW, and the lack of disaggregated data has led to a paucity of diabetes data among Native Hawaiians or Pacific Islanders^(4–6)^. These statistics indicate that Asian Americans and Native Hawaiians or Pacific Islanders (AAPI) are less visible and more vulnerable to diabetes and related complications^(1,3)^.

While food access has been discussed as a determinant of diabetes, evidence on its associations with diabetes in Asian countries (given the limited studies specifically on AAPI) and the USA remains mixed^(7–9)^. In India, individuals’ fasting glucose showed a negative association with fruit and vegetable vendor density within a 400-m buffer, and a weak positive association with processed food vendor density within a 400-m buffer^(10)^. A study in Thailand found no association between diabetes and individuals’ shopping patterns at supermarkets or convenience stores^(11)^. In the USA, some studies indicated a positive association between diabetes and the relative availability of fast-food restaurants, and a negative association with the relative availability of supermarkets, while others found no associations between diabetes and either outlet in high-minority neighbourhoods^(12–14)^.

Inconsistencies in the current literature may stem from an oversimplification of food access or a lack of consideration of sociocultural and economic norms in food consumption^(7)^. Current literature on food outlets and nutrition environments mainly focuses on supermarkets, convenience stores, fast-food restaurants or full-service restaurants^(15)^. However, ethnic stores, which are important sources of fresh, healthy and culturally appropriate food preferred by racial and ethnic minorities, have rarely been studied^(15,16)^. Emerging studies have shown that while there is a lower concentration of supermarkets in communities of colour, racial and ethnic minorities may prefer to shop at ethnic grocery retailers due to their sense of identity and cultural affinity^(15,17–19)^. A study among Chinese Americans in New York, NY, has demonstrated that immigrants prioritise culturally specific food over distance or convenience of grocery shopping, as individuals travel 1·5 miles further for ethnic stores on average^(16)^. The Nielsen Asian American Consumer Report also showed that Asian American households purchased 182 % more dry vegetables and grains, 69 % more fresh seafood and 72 % more fresh vegetables than the overall US population, suggesting potential distinct patterns in AAPI’s food shopping preferences^(20)^. Despite the cultural and nutritional significance of ethnic stores, there is a lack of population-level tools to measure access to culturally appropriate food. Many studies rely on the Food Access Research Atlas (FARA) developed by the US Department of Agriculture (USDA), which is a food desert map at the census tract (CT) level based on supermarket access for the CT population^(21–23)^. Other studies have used store audits to measure culturally appropriate food access, which, due to their time- and labour-intensive nature, can pose challenges for large-scale measurement^(15,24)^. Not accounting for the nutritional and food shopping preferences of racial and ethnic minorities may result in a misrepresentation of the association between food access and chronic health conditions like diabetes^(15)^.

Area-level, culturally sensitive measures of food access are not currently used in food environment and health research. Thus, this ecological study examines the relationship between access to culturally appropriate food and diabetes rates among AAPI in San Diego (SD) County, CA. It addresses research gaps by (1) developing the Yelp-based Ethnic Store Measure – a population-level indicator of access to culturally appropriate food using six novel variables derived from crowd-sourced Yelp data, (2) exploring how FARA and Yelp-based Ethnic Store Measure relate to AAPI diabetes at the CT level and (3) comparing the associations of FARA and Yelp-based Ethnic Store Measure with AAPI diabetes. We hypothesise that our Yelp-based Ethnic Store Measure, which considers cultural preferences, will be more strongly associated with AAPI rates of diabetes at the CT level than the FARA measure of food access.

## Methods

### Food access data

#### Food Access Research Atlas

This study was conducted in San Diego County, CA, at the CT level using 2010 geographies, which aligned with the areal zoning system of USDA’s 2019 FARA dataset. FARA measures food access based on the distance to the nearest supermarket for the total population in each CT and for subpopulations defined by race and ethnicity, income and age^(23)^. We used the estimates of the share of total CT population that is beyond ½ mile of a supermarket (FARA_CT). We also combined estimates of Asian Americans with those of Native Hawaiians or Pacific Islanders to calculate the share of CT’s AAPI population beyond ½ mile of a supermarket (FARA_AAPI). We recognise the diversity of ethnicities represented in the AAPI population and have provided details on the AAPI ethnic composition in San Diego in 2020 (Appendix A, Table A1).

#### Yelp dataset

Yelp is a crowd-sourced review website used across the USA, and it has been used to measure healthy food access and its association with cardiometabolic diseases^(25–27)^. The Yelp business registry data for San Diego County was extracted from Yelp.com in December 2020 using the Yelp Fusion API^(28)^. Only businesses that were classified under the restaurant, food and shopping categories per the Yelp business category list were collected into our registry dataset^(29)^. Attributes of each business in the Yelp registry data included the business name, category, rating, business location and URL of the business’s Yelp page. Point locations were in the form of x and y coordinates, so they did not need geocoding.

To make the Yelp-based Ethnic Store Measure comparable and complementary to the FARA variables, this study first categorised food outlets based on type (‘restaurant’, ‘grocery’ or ‘neither’) and then grouped them as majority-AAPI-food (hereinafter ‘AAPI’) or non-majority-AAPI-food (hereinafter ‘non-AAPI’). Classification began at the Yelp category level, with additional reviews of food outlets within each category. Outlets labelled with categories that were not food-related (e.g. bookstores) or that lacked physical or consistent locations (e.g. food delivery services) were excluded (Appendix A, Yelp Business Details). All outlets with categories under the ‘Restaurant’ list on the Yelp website were grouped as ‘restaurant’^(29)^. Outlets with categories that contained ‘grocery’, ‘farmer’s market’, ‘food market’ and ‘health market’ were grouped as ‘grocery’. The rest were grouped as ‘neither’.

To classify categories as AAPI or non-AAPI, they were reviewed independently by two researchers and classified into three groups: ‘AAPI’, ‘non-AAPI’ and ‘ambiguous’. Discrepancies between the two researchers were addressed by consulting with the senior author. Categories in the ‘AAPI’ group included those with specific Asia-Pacific region identifiers (e.g. ‘Chinese’, ‘Hawaiian’ and ‘Asian Fusion’) and foods originating from Asia or the Pacific Islands (e.g. ‘Bubble Tea’, ‘Poke’ and ‘Ramen’). Categories in the ‘ambiguous’ group included general terms that may encompass AAPI food (e.g. ‘Herbs & Spices’ and ‘Grocery’). The remaining categories were placed in the ‘non-AAPI’ group, including those with specific non-Asia-Pacific identifiers (e.g. ‘Mexican’ and ‘Traditional American’) and foods not originating from Asia or the Pacific Islands (e.g. ‘Soul Food’ and ‘Beer Bar’). Outlets in the ‘ambiguous’ group were searched online (e.g. Google Maps, Yelp) by three researchers. The researchers reviewed photos and menu items of the outlets together, assessed whether the outlets primarily sold AAPI food and determined whether the outlets were ‘AAPI’ or ‘non-AAPI’.

**Figure 1.**
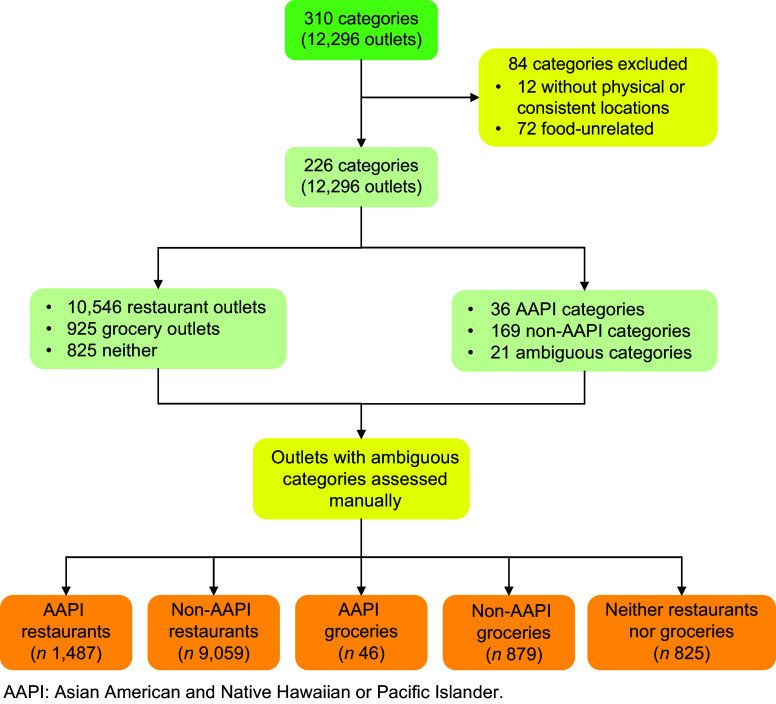
Coding of food outlets from the Yelp dataset. AAPI, Asian American and Native Hawaiian or Pacific Islander.

A list of 12 296 food outlets across 310 categories was obtained from Yelp. Using the process detailed in Figure 1, all outlets in the dataset were sorted into five mutually exclusive groups based on type (‘restaurant’, ‘grocery’ or ‘neither’) and content (‘AAPI’ or ‘non-AAPI’): (1) AAPI restaurants, (2) non-AAPI restaurants, (3) AAPI groceries, (4) non-AAPI groceries and (5) neither restaurants nor grocery stores.

#### Yelp-based Ethnic Store Measure

From the categorised Yelp data, variables of the Yelp-based Ethnic Store Measure were calculated at the CT level. We explored six different Yelp-based variables to summarise three aspects of ethnic food access: (1) relative food environment, which measures the AAPI food opportunities relative to the total food environment within CT, (2) outlets per capita, which measures the proximity between food outlets and CT populations and (3) outlet intensity, which measures CT’s AAPI food opportunities relative to the county total. These three aspects were each represented by two variables (Appendix A, Table A2). For the relative food environment, we calculated the ratio of AAPI restaurants to non-AAPI restaurants (AAPI restaurant ratio) and the ratio of AAPI groceries to non-AAPI groceries (AAPI grocery ratio) within each CT. For outlets per capita, we divided the numbers of AAPI restaurants and AAPI groceries by the AAPI population in CT (AAPI restaurants per AAPI and AAPI groceries per AAPI). For the outlet intensity, we divided the numbers of AAPI restaurants and AAPI groceries in each CT by the county total of AAPI restaurants and AAPI groceries (AAPI restaurant intensity and AAPI grocery intensity). We used the Geographic Information Systems Food Environment ReportiNg (Geo-FERN) checklist to ensure that the reporting of methodological choices in the creation of these food environment measures was clear and consistent with best practices (Appendix B, Geo-FERN Checklist)^(30)^.

### Diabetes rate

Estimated diabetes rates were based on reported AAPI hospitalisations and emergency department discharges for diabetes from the San Diego County Health & Human Services Agency^(31)^. The most granular level of the diabetes data documented estimates at the Subregional Area (SRA) level of diabetes discharge case counts for Asian Americans and Native Hawaiians or Pacific Islanders combined. The SRA diabetes discharge case counts for AAPI were first averaged for 2011–2017 (the most recent available) to obtain the annual SRA discharge diabetes case counts, which were then divided by the SRA AAPI population to get the SRA annual discharge diabetes rate per 100 000 AAPI population. We utilised this measure as a proxy for AAPI diabetes rates^(32)^.

The annual AAPI diabetes rates at the SRA geography were then downscaled to CT for finer geographic analysis and to match food access variables and covariates. To obtain annual CT rates of AAPI diabetes, we apportioned the annual SRA diabetes rates per 100 000 population to CT based on the proportion of AAPI in the CT to the total AAPI in the SRA. CT with 0 AAPI population were assigned 0 for diabetes rates (*n* 4). In some SRA (*n* 21), AAPI diabetes data were suppressed due to small numbers of AAPI and were assigned null. To assign diabetes data to these SRA and their constituent CT, we first took the difference in total AAPI diabetes reported for the entire county (*n* 3530) and the total reported AAPI diabetes in SRA (*n* 3150), which was 380 unapportioned AAPI diabetes cases. These 380 AAPI cases were then converted into annual rates per 100 000 AAPI population and apportioned among the twenty-one null SRA based on the proportion of AAPI in each SRA of the null set of SRA. The SRA’s AAPI diabetes rates were then apportioned to the CT using the same method above, based on the proportion of AAPI in the CT within the SRA.

### Covariates

Sociodemographic data were obtained from the 2019 American Community Survey 5-year estimates, including race and ethnicity, percentage of CT population below 200 % federal poverty line and percentage of CT population over 65 years old^(33–35)^. We also obtained CT obesity estimates from the CDC PLACES database, which included small-area, model-based estimates for various health behaviours and outcomes^(36)^. Lastly, we assessed population density, calculated as the total CT population divided by the area of residential land use, with data from San Diego’s Regional Planning Agency land use database^(35,37)^.

### Statistical analysis

To compare FARA with Yelp-based Ethnic Store Measure, we mapped FARA and Yelp-based variables and examined them for general spatial trends using Moran’s *I* with k-nearest neighbours. Spearman’s correlation was performed to test the associations among the FARA and Yelp-based variables. Variables with high correlation coefficients (> 0·90) were dropped from further regression modelling.

We used ordinary least squares (OLS) regression to assess global associations and spatial lag regression (SLR) to account for spatial dependency in the dependent variable. Spatial autocorrelation of OLS model residuals was assessed for significant spatial dependence. OLS and SLR models assessed covariate-adjusted associations between food access variables and CT’s AAPI diabetes rates, with food access variables and covariates standardised to z-scores. Covariates included population density, percentage of CT population with obesity and percentage over 65 years old. While socio-economic status is often a predictor of diabetes, it was not included as a covariate in our models, as CT-level poverty was included as a predictor in the PLACES model-based estimates of obesity. Geospatial mapping and analyses used ArcGIS 10.8 (ESRI), and statistical analyses used R 4.3.1 (The R Foundation for Statistical Computing). Shapefile reading used *maptools* package, and SLR models used *lagsarlm* function under *spdep* package, with queen contiguity as the spatial neighbour measurement type. *P* < 0·05 was used to determine statistical significance. All data and statistical analytic code utilised in this study are available at https://github.com/hdscalecollab/aapi-food-access-sd.

## Results

### Spatial patterns of Food Access Research Atlas and Yelp-based Ethnic Store Measure

Depending on the variable, data were available from 624 (99·68 %) to 626 (100 %) CT in San Diego County except for the two FARA variables, which were available in 564 (90·10 %) CT. Most CT without FARA data were clustered around the southwestern part of the county, with a few located in the central north part of the county. Based on the 2019 ACS 5-year estimates, CT without FARA data constituted 8·87 % of the total population in San Diego County^(33)^. Table 1 compares the health outcomes and sociodemographics between CT with and without FARA data. No significant differences were observed in AAPI diabetes and percent AAPI, but the remaining three variables showed significant differences: Compared to CT with FARA data, CT without FARA data had a lower percentage of population over 65 years old, a higher percentage of obesity and a higher population density.

**Table 1.** Comparison of census tracts (CT) with and without Food Access Research Atlas (FARA) data

Variable	CT with FARA data (*n* 564)		CT without FARA data (*n* 62)		*P*
	Mean	sd	Mean	sd	
AAPI diabetes	6·17	12·89	7·72	13·52	0·39
Percent AAPI	10·87	11·20	10·64	12·13	0·88
Percent over 65 years old	14·92	7·58	9·91	4·69	< 0·001
Percent obesity	23·05	3·52	25·93	4·47	< 0·001
Population density	17 219	21 482	41 206	35 417	< 0·001

AAPI, Asian American and Native Hawaiian or Pacific Islander.

**Figure 2.**
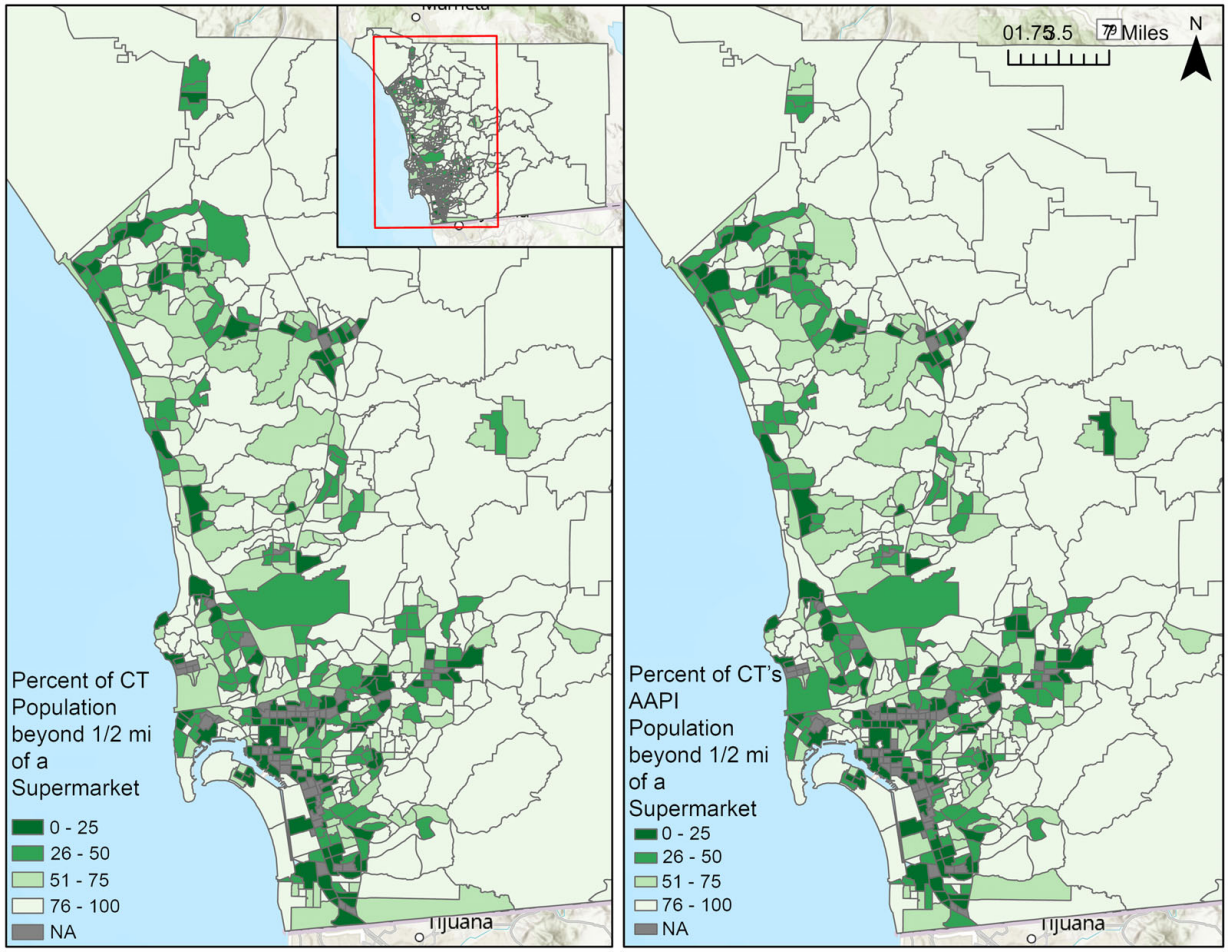
Food Access Research Atlas (FARA) variables: the percent of census tract (CT) population beyond ½ mile from a supermarket (FARA_CT, left), and the percent of CT’s Asian American and Native Hawaiian or Pacific Islander (AAPI) population beyond ½ mile from a supermarket (FARA_AAPI, right).

Figure 2 visualises the percent of CT population beyond ½ mile of a supermarket (FARA_CT), and the percent of CT’s AAPI population beyond ½ mile of a supermarket (FARA_AAPI). On average, 58·9 % of the total population and 58·11 % of the AAPI population in an SD CT were beyond ½ mile from a supermarket. More than one-fifth of CT had over 95 % of both the total population and the AAPI population beyond ½ mile from a supermarket. For both FARA variables, CT in coastal and southwestern SD had higher supermarket access and lower percentages beyond ½ mile of a supermarket.

**Figure 3.**
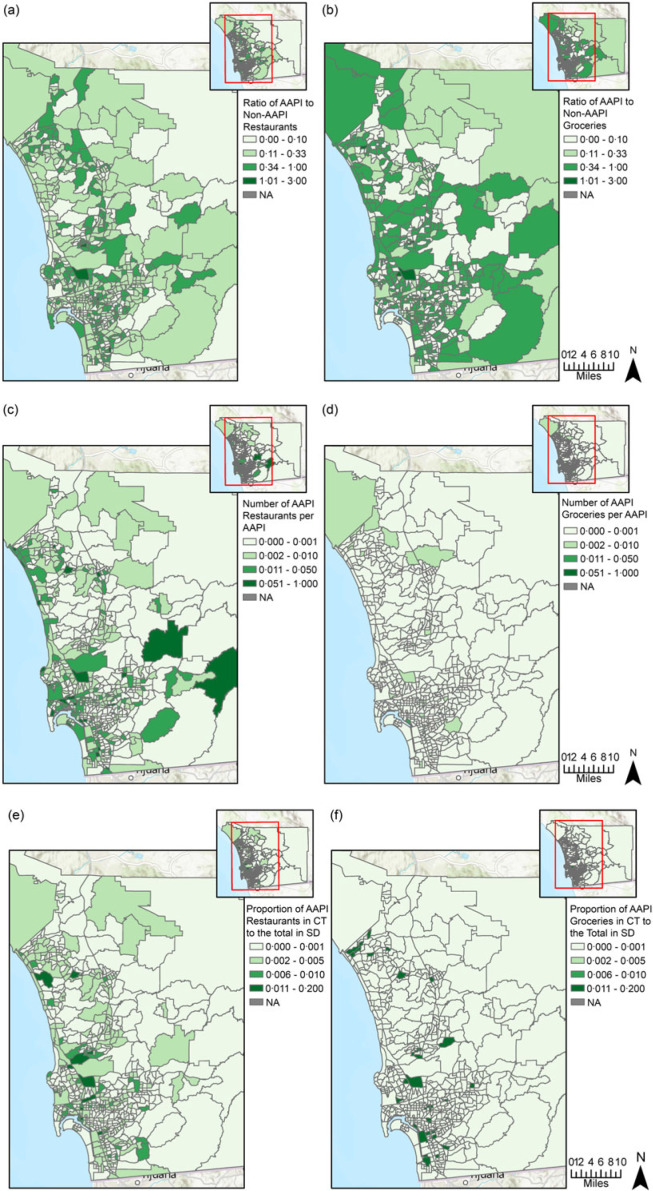
From (a) to (f), Yelp-based variables of Asian American and Native Hawaiian or Pacific Islander (AAPI) food access: (a) ratio of AAPI to non-AAPI restaurants; (b) ratio of AAPI to non-AAPI grocery stores; (c) ratio of AAPI restaurants to AAPI population; (d) ratio of AAPI grocery stores to AAPI population; (e) proportion of census tract (CT) AAPI restaurants to the total number of AAPI restaurants in San Diego County; (f) proportion of CT AAPI grocery stores to the total number of AAPI grocery stores in San Diego County.

Figure 3 delineates the spatial distribution of the six Yelp-based Ethnic Store variables. For every non-AAPI restaurant and non-AAPI grocery store in CT, there was an average of 0·24 AAPI restaurants and 0·26 AAPI grocery stores, respectively. For every 1000 AAPI population in CT, there was an average of 9 AAPI restaurants and 0·2 AAPI grocery stores.

For both restaurants and groceries, coastal areas of SD had a greater ratio of AAPI to non-AAPI outlets, a higher number of AAPI outlets per AAPI and a higher proportion of AAPI outlets over the county total than inland areas. However, compared to AAPI groceries, AAPI restaurants had a larger volume and were more geographically dispersed in terms of per capita count and intensity (Figure 3(c)–(f)).

### Covariate-adjusted models

#### Ordinary least squares regression

In terms of diabetes rate and sociodemographics, CT in SD had an average of 6·33 diabetes discharges per 100 000 AAPI population per year. On average, 10·85 % of the population in CT identified as AAPI, 14·42 % were over 65 years old and 23·33 % were obese. We dropped two food access variables – FARA_CT and AAPI restaurant intensity – from the analyses due to their significant correlations with FARA_AAPI and AAPI restaurants per AAPI, respectively (Appendix A, Table A3). The modelling focused on the six remaining food access variables: FARA_AAPI, AAPI restaurant ratio, AAPI restaurants per AAPI, AAPI grocery ratio, AAPI groceries per AAPI and AAPI grocery intensity.

Table 2 shows that in OLS models adjusting for CT’s population density, percent obesity and percent over 65 years old, a 1-sd increase in the share of CT’s AAPI population beyond ½ mile of a supermarket was associated with a 1·88-unit increase in CT’s AAPI diabetes rate (95 % CI 0·76, 3·00) (*P* = 0·001). A 1-sd increase in CT’s number of AAPI restaurants per AAPI population was associated with a 1·15-unit decrease in CT’s AAPI diabetes rate (95 % CI –2·17, –0·13) (*P* = 0·03). Associations between the four other food access variables and AAPI diabetes were not significant. Spatial clustering for residuals of all OLS models was significant at *P* < 0·001 under both k-nearest neighbours (k = 8) and queen contiguity. This suggested the CI and *P*-values of OLS models were biased and had to be interpreted with caution.

**Table 2.** Ordinary least squares (OLS) regression for Food Access Research Atlas (FARA) and Yelp-based variables (outcome: census tract rate of AAPI diabetes hospitalisations and ED discharges per 100 000 AAPI population per year)

Food access variable	*n*	Standardised est	Standardised 95 % CI	*P*
FARA_AAPI	564	1·88	0·76, 3·00	0·001
AAPI restaurant ratio	626	0·38	−0·63, 1·40	0·46
AAPI restaurants per AAPI	624	−1·15	−2·17, –0·13	0·03
AAPI grocery ratio	626	0·60	−0·42, 1·62	0·25
AAPI groceries per AAPI	626	−0·03	−1·05, 0·99	0·95
AAPI grocery intensity	626	0·58	−0·44, 1·60	0·27

AAPI, Asian American and Native Hawaiian or Pacific Islander; ED, emergency department; FARA_AAPI, percent of CT’s AAPI population > ½ mile to the nearest supermarket.

Adjusted for population density, obesity and age.

#### Spatial lag regression

Four of the six food access variables – FARA_AAPI, AAPI restaurant ratio, AAPI grocery ratio and AAPI grocery intensity – showed significant spatial dependence at *P* < 0·05, indicating the need for SLR models to account for spatial autocorrelation (Appendix 4, Table A4). Table 3 shows that in SLR models adjusting for CT’s population density, percent obesity and percent over 65 years old, a 1-sd increase in CT’s number of AAPI restaurants per AAPI population was associated with a 1·32-unit decrease in AAPI diabetes rate in the CT (*P* = 0·004). It also had the smallest AIC and the highest log-likelihood among all SLR models. Although FARA_AAPI was significant in OLS, FARA_AAPI and four Yelp-based variables were not significant in SLR. No model indicated spatial autocorrelation in the SLR residuals under both k-nearest neighbours (k = 8) and queen contiguity.

**Table 3.** Spatial lag regression (SLR) for Food Access Research Atlas (FARA) and Yelp-based variables (outcome: census tract rate of AAPI diabetes hospitalisations and ED discharges per 100 000 AAPI population per year)

Food access variable	Standardised est	*P*	Rho	Log-likelihood	AIC
FARA_AAPI	0·66	0·18	0·552	−2427	4868
AAPI restaurant ratio	0·16	0·72	0·559	−2428	4870
AAPI restaurants per AAPI	−1·32	0·004	0·566	−2424	4862
AAPI grocery ratio	0·68	0·13	0·561	−2427	4868
AAPI groceries per AAPI	−0·03	0·94	0·559	−2428	4870
AAPI grocery intensity	0·27	0·56	0·558	−2428	4870

AAPI, Asian American and Native Hawaiian or Pacific Islander; ED, emergency department; AIC, Akaike Information Criterion; FARA_AAPI, percent of CT’s AAPI population > ½ mile to the nearest supermarket.

Adjusted for population density, obesity and age.

## Discussion

This study developed the Yelp-based Ethnic Store Measure, a novel measure of AAPI’s access to culturally appropriate food based on the Yelp dataset, and applied it in comparison to FARA to investigate their respective covariate-adjusted relationship with AAPI diabetes rate in San Diego, CA. Both FARA and the Yelp-based Measure displayed higher food access in the coastal and southwest areas, but the Yelp-based Measure showed a more dispersed distribution of CT with medium-to-high access to culturally appropriate food. The SLR model was the more appropriate method for assessing associations between food access variables and diabetes, given the presence of spatial autocorrelation in four of the six food access variables and all OLS model residuals. Our hypothesis that our Yelp-based Ethnic Store Measure would be more strongly associated with AAPI diabetes rates than FARA partially holds true, as one of the Yelp-based variables, the number of AAPI restaurants per AAPI population, showed stronger associations with AAPI diabetes rates than FARA. This is a key contribution to support the usefulness of area-level, culturally sensitive measures of food access and health outcomes.

Our study demonstrated that the ability of the FARA map to delineate food access must be carefully considered while studying minority health outcomes related to access to culturally appropriate food. In the existing literature on food outlets with different healthfulness, there can be a risk of falling into the binary narratives of healthy *v*. unhealthy: supermarket *v*. convenience store and full-service *v*. fast-food restaurants^(15,38)^. FARA, with its exclusive focus on supermarket access, can be one example of food environment measures that reinforce this binary. By overlooking the existing networks of culturally appropriate outlets in minority communities, FARA’s sole focus on supermarkets can potentially misrepresent the association between food access and diabetes among racial and ethnic minorities^(15)^. Our Yelp-based Ethnic Store Measure includes food outlets that cater to specific ethnicities or cultural preferences. Thus, compared to FARA, the Yelp-based Ethnic Store Measure provides additional information on small ethnic outlets, or the ‘in-betweens’ that serve as an important source of fresh, healthy and culturally appropriate food and that align with the preferences and cultural contexts of racial and ethnic minorities^(15,38)^.

When comparing FARA with the Yelp-based Measure, FARA was missing data in 9·90 % of CT in SD, which could bias associations if it acts as a proxy for food access. While FARA showed that a lack of supermarket access was a risk factor for diabetes in the OLS model, it was not significant after adjusting for spatial autocorrelation in the SLR model. As one of the nation’s few population-level measures of food access, FARA has been widely used for policymaking and monitoring. However, as this study found, FARA has limited capacity to represent the population’s access to culturally appropriate food or capture the connection between food access and chronic health outcomes. This could lead to potential misguidance and unintended consequences of food policies and public health interventions, especially for racial and ethnic minorities who are already disproportionately impacted by disparate health outcomes^(39)^.

Based on our Yelp-based Ethnic Store Measure, this study found that a higher number of AAPI restaurants per AAPI population was associated with a lower neighbourhood-level AAPI diabetes rate. This finding is consistent with a prior San Diego study that highlighted the contribution of ethnic stores to local food security and residents’ access to nutritious, affordable and culturally acceptable food items^(15)^. One possible explanation for the reported negative association between AAPI restaurants and diabetes can be the health implications of acculturation, which is the adaptation of nutritional practices to the food availability of the country one lives in^(40)^. When there is a lack of access to culturally appropriate food, individuals might involuntarily adopt less ethnically traditional diets or highly processed versions of their ethnic dishes from mainstream grocery stores, thereby increasing the risks for cardiometabolic diseases^(40)^. Past research has shown correlations between acculturation and higher intakes of Na, fat, convenience foods and sugar-sweetened beverages, as well as poorer self-reported health status among Asian immigrants in North America^(40–42)^.

Another possible explanation is that living in co-ethnic neighbourhoods, where there are more ethnic restaurants, may positively impact the health of racial and ethnic minorities. A previous study has found that ‘living in a tract with a higher proportion of immigrants was associated with lower consumption of high-fat foods among Hispanics and Chinese’ and ‘better healthy food availability’^(43)^. To explore this further, we examined the Pearson correlation between percent AAPI in CT and Yelp-based variables. The correlation coefficient between percent AAPI and AAPI restaurant intensity was 0·13 (*P* = 0·001), indicating a statistically significant but weak positive association. On the other hand, the number of AAPI restaurants per AAPI – the Yelp-based variable that was negatively associated with AAPI diabetes rates – had a weak negative correlation with percent AAPI in CT (*r* = –0·17, *P* < 0·001). In other words, CT with higher AAPI population density do not necessarily have more AAPI restaurants per AAPI. This may suggest that the negative association between AAPI restaurants per AAPI and diabetes may not be driven solely by co-ethnic community presence, but potentially by other mechanisms such as broader food environment exposure or community integration. By introducing an innovative measure of access to culturally appropriate food, our study offers valuable insights into its role as an underlying environmental determinant of cardiometabolic health among racial and ethnic minorities.

There are several limitations in our study. First, when calculating the Yelp-based variables, the classification of Yelp categories and food outlets was done manually, albeit expedited by a standardised procedure. Thus, there may be possibilities of human error, and the process can be time-consuming for future replication. Second, the aggregation of data for AAPI into a single AAPI population group overlooks the heterogeneity and health disparities within the AAPI population, since AAPI ethnic subgroups differ in prevalence rates of diabetes and foodways^(44,45)^. While lumping diverse ethnic groups into one AAPI category is not ideal, this study was limited by the unavailability of diabetes data for AAPI subgroups, as our diabetes case count data from San Diego County combined estimates for Asian Americans with those for Native Hawaiians or Pacific Islanders. Third, due to the lack of CT-level diabetes data, this study calculated the CT diabetes estimates based on the SRA-level data, assuming that the diabetes rate was constant across the SRA. It is unclear how this assumption relates to the reported results; however, this was based on a limited number of SRA. We also acknowledge that our outcome variable, limited to hospitalisations and emergency department discharges over a 7-year period, captured only a subset of individuals with diabetes; however, it served as the most feasible proxy for estimating diabetes rates. Lastly, we had CT’s sociodemographics as well as the small-area, model-based obesity estimates from the CDC PLACES database as our covariates. Thus, the significant associations can be due to the post-stratification estimation process, which generated model estimates based on the same demographics we included as covariates. This might result in associations that reflect the relationship between obesity estimates and sociodemographic covariates, instead of between independent and dependent variables^(46)^.

Our findings underscore the usefulness of a more nuanced understanding of the many types of food outlets that comprise the food environment, which improves upon existing food access and environment research assessing the lack of supermarkets to define food deserts. Future research could develop adaptations of our Yelp-based Ethnic Store Measure or advance other ethnic food measures to cater to the needs of different subpopulations. Data disaggregation is also needed for more suitable and accurate food access measures. Lastly, research on the nutrition environment of AAPI restaurants could be conducted to more closely investigate the underlying mechanisms of the correlation between AAPI restaurants and diabetes that was reported in this study.

## Supporting information

Xie et al. supplementary material 1 Xie et al. supplementary material

Xie et al. supplementary material 2 Xie et al. supplementary material

## Supplementary material

For supplementary material accompanying this paper visit https://doi.org/10.1017/S1368980025101444
