# Supplementary material for: Measuring access to culturally appropriate food and associations with diabetes among Asian Americans and Native Hawaiians or Pacific Islanders
Source: Public Health Nutr. 2025 Nov 12;28(1):e197. doi: 10.1017/S1368980025101444 (PMC12722082; doi:10.1017/S1368980025101444)
Supplement: Xie et al. supplementary material 1 — Xie et al. supplementary material [file S1368980025101444sup001.docx]

**Appendix A**

Table A1: Ethnic composition of the AAPI population in San Diego in 2020

| Ethnic Group | Percent of Total AAPI Population (%) |
| --- | --- |
| **Central Asian** (Kazakh, Uzbek, or have other Central Asian ancestry) | 0.7 |
| **East Asian** (Chinese, Korean, Japanese, Hmong, Okinawan, Taiwanese, Mongolian, or have other East Asian ancestry) | 31.4 |
| Chinese | 16.3 |
| Korean | 5.9 |
| Japanese | 7.5 |
| **Native Hawaiian or Other Pacific Islander** | 1.0 |
| **South Asian** (Indian, Pakistani, Bangladeshi, Nepalese, Sri Lankan, Bhutanese, Sikh, or have other South Asian ancestry) | 10.5 |
| Indian | 9.6 |
| **Southeast Asian** (Filipino, Vietnamese, Cambodian, Laotian, Thai, Burmese, Indonesian, Malaysian, Mien, Singaporean, or have other Southeast Asian ancestry) | 55.3 |
| Filipino | 38.6 |
| Vietnamese | 11.2 |

AAPI, Asian Americans and Native Hawaiians or Pacific Islanders.

Note: percents do not equal 100 due to rounding, multiple race/ethnicity categories, or suppression of small numbers

Source: <https://healthatlas.ucsf.edu/>

Yelp Business Details

To make sure that only food establishments with actual and consistent locations were included in the analysis, the exclusion criteria in the Yelp-based analysis were:

1. Businesses with any of the following labels (n=12):

| Aquariums | Food Stands | Pop Up Shops | Vitamins Supplements |
| --- | --- | --- | --- |
| Beer Tours | Food Tours | Pop-Up Restaurants | Weight Loss Centers |
| Food Delivery Services | Food Trucks | Street Vendors | Wine Tours |

1. Businesses that had any of the following labels and didn’t sell food or produce (n=72):

| Activities | Drug Stores | Limos | Shopping Centers |
| --- | --- | --- | --- |
| Amusement Parks | Dumpster Rental | Movie Theaters | Souvenirs |
| Arcades | Entertainment | Museums | Specialty Schools |
| Art Classes | Electronics | Music Venues | Stationery |
| Axe Throwing | Event Planning | Nonprofit | Swimming Pools |
| Book Stores | Festivals | Nutritionists | Tasting Classes |
| Bowling | Fitness | Paint Your Own Pottery | Theater |
| Brewing Supplies | Flea Markets | Party Equipment Rentals | Tobacco Shops |
| Casinos | Florists | Party Supplies | Toys |
| Cigar Bars | Gift Shops | Pet Adoption | Truck |
| Comedy Clubs | Golf | Pharmacy | Vape Shops |
| Comic Books | Hardware | Play Center | Venues |
| Cooking Classes | Head Shops | Pool Halls | Veterans Organizations |
| Cooking Schools | Home And Garden | Print Media | Water Stores |
| Cosmetics | Home Decor | Public Markets | Wine Taste Classes |
| Country Dance Halls | Hotels | Recycling Center | Wine Tasting Room |
| Couriers | Jazz And Blues | Rental | Womens Cloth |
| Dance Clubs | Karaoke | Shopping | Yoga |

Table A2: Variables in the Yelp-based Ethnic Store Measure

| Group | Yelp Variable | Calculation |
| --- | --- | --- |
| 1. Relative food environment:  Ratio of AAPI to non-AAPI food outlets within CT | AAPI restaurant ratio | $\frac{\# AAPI restaurants in CT}{\# Non-AAPI restaurants in CT}$ |
|  | AAPI grocery ratio | $\frac{\# AAPI groceries in CT}{\# Non-AAPI groceries in CT}$ |
| 2. Outlets per capita:  Ratio of AAPI food outlets to AAPI population in CT | AAPI restaurant per AAPI | $\frac{\# AAPI restaurants in CT}{\# AAPI population in CT}$ |
|  | AAPI grocery per AAPI | $\frac{\# AAPI groceries in CT}{\# AAPI population in CT}$ |
| 3. Outlet intensity:  Proportion of AAPI food outlets in CT to total AAPI food outlets in San Diego County | AAPI restaurant intensity | $\frac{\# AAPI restaurants in CT}{\# AAPI restaurants in County}$ |
|  | AAPI grocery intensity | $\frac{\# AAPI groceries in CT}{\# AAPI groceries in County}$ |

AAPI, Asian Americans and Native Hawaiians or Pacific Islanders. CT, census tract.

Table A3: Variables removed from modeling

| Removed variables | Kept variables | Spearman’s correlation coefficient ρ | P |
| --- | --- | --- | --- |
| FARA_CT | FARA_AAPI | .98 | <.001 |
| AAPI restaurant intensity | AAPI restaurant per AAPI | .91 | <.001 |

FARA_AAPI, percent of CT’s AAPI population >½ mile to the nearest supermarket. FARA_CT, percent of CT population >½ mile to the nearest supermarket. AAPI, Asian Americans and Native Hawaiians or Pacific Islanders.

Table A4: Spatial autocorrelation analysis of food access variables using k-nearest neighbors (k=8) and queen contiguity

|  |  | FARA_AAPI | AAPI restaurant ratio | | AAPI restaurant per AAPI | AAPI grocery ratio | AAPI grocery per AAPI | AAPI grocery intensity |
| --- | --- | --- | --- | --- | --- | --- | --- | --- |
| K-nearest neighbors (k=8) | p-value | <.001 | <.001 | .20 | | .02 | .74 | <.001 |
|  | z score | 10.73 | 7.39 | 1.27 | | 2.38 | -.33 | 3.71 |
| Queen contiguity | p-value | <.001 | <.001 | .17 | | .08 | .88 | .008 |
|  | z score | 9.96 | 6.34 | 1.39 | | 1.75 | .15 | 2.66 |

FARA_AAPI, percent of CT’s AAPI population >½ mile to the nearest supermarket. AAPI, Asian Americans and Native Hawaiians or Pacific Islanders.
